# New insights into the origin of the B genome of hexaploid wheat: Evolutionary relationships at the *SPA *genomic region with the S genome of the diploid relative *Aegilops speltoides*

**DOI:** 10.1186/1471-2164-9-555

**Published:** 2008-11-25

**Authors:** Jérome Salse, Véronique Chagué, Stéphanie Bolot, Ghislaine Magdelenat, Cécile Huneau, Caroline Pont, Harry Belcram, Arnaud Couloux, Soazic Gardais, Aurélie Evrard, Béatrice Segurens, Mathieu Charles, Catherine Ravel, Sylvie Samain, Gilles Charmet, Nathalie Boudet, Boulos Chalhoub

**Affiliations:** 1 UMR INRA 1165 – CNRS 8114 UEVE – Unité de Recherche en Génomique Végétale (URGV), 2, rue Gaston Crémieux, CP5708, 91057 Evry cedex, France; 2 UMR 1095 INRA – Université Blaise Pascal – Génétique Diversité Ecophysiologie de Céréales (GDEC), Domaine de Crouelle, 234, avenue du Brézet, F-63100, Clermont-Ferrand, France; 3 CEA: Institut de génomique – GENOSCOPE, 2, rue Gaston Crémieux, CP 5706, 91057, EVRY Cedex, France

## Abstract

**Background:**

Several studies suggested that the diploid ancestor of the B genome of tetraploid and hexaploid wheat species belongs to the *Sitopsis *section, having *Aegilops speltoides *(SS, 2n = 14) as the closest identified relative. However molecular relationships based on genomic sequence comparison, including both coding and non-coding DNA, have never been investigated. In an attempt to clarify these relationships, we compared, in this study, sequences of the Storage Protein Activator (SPA) locus region of the S genome of *Ae. speltoides *(2n = 14) to that of the A, B and D genomes co-resident in the hexaploid wheat species (*Triticum aestivum, AABBDD*, 2n = 42).

**Results:**

Four BAC clones, spanning the SPA locus of respectively the A, B, D and S genomes, were isolated and sequenced. Orthologous genomic regions were identified as delimited by shared non-transposable elements and non-coding sequences surrounding the SPA gene and correspond to 35 268, 22 739, 43 397 and 53 919 bp for the A, B, D and S genomes, respectively. Sequence length discrepancies within and outside the SPA orthologous regions are the result of non-shared transposable elements (TE) insertions, all of which inserted after the progenitors of the four genomes divergence.

**Conclusion:**

On the basis of conserved sequence length as well as identity of the shared non-TE regions and the SPA coding sequence, *Ae speltoides *appears to be more evolutionary related to the B genome of *T. aestivum *than the A and D genomes. However, the differential insertions of TEs, none of which are conserved between the two genomes led to the conclusion that the S genome of *Ae. speltoides *has diverged very early from the progenitor of the B genome which remains to be identified.

## Background

All cereal crop species are members of the grass (*Poaceae*) family that is the fourth largest family of flowering plants. With about 10 000 species growing under nearly all climates and latitudes, grasses exceed all other plant families in ecological dominance and economic importance. In terms of genome organisation they represent a very diverse family with basic chromosome numbers ranging from 4 to 50 and genome sizes ranging from 350 Mb to 17 Gb [1]. Fossil data and phylogenetic studies have estimated that the grasses have diverged from a common ancestor 50 to 70 million years ago (MYA) [2,3]. Archaeological records suggest that farming started concomitantly in at least three widely separated regions between 10 000-5 000 years ago during the late Neolithic period. The three most important cereals were independently domesticated in three centres: wheat in south western Asia in the 'Fertile Crescent' region, maize in Mexico and rice in both south east Asia and west Africa [4-6].

Within the *Poaceae*, the genera *Aegilops *and *Triticum *include several diploid species (2n = 14) that, via allopolyploidization, produced several tetraploid and hexaploid wheat species, most of which have been domesticated [7-9]. *T. turgidum *(2n = 28, AABB) was derived from a hybridization event that happened (< 0.5 MYA) between *T. urartu*, (2n = 14, AA), the diploid donor of the A genome (here after gA), and another unknown species of the *Sitopsis *section, donor of the B genome (here after gB), for which the closest known relative is *Ae. speltoides *[7,9,10]. The hexaploid wheat (*T. aestivum*, 2n = 42, AABBDD) originated from an additional polyploidization event between the early-domesticated tetraploid *T. turgidum ssp dicoccum *and the diploid donor of the D genome (here after gD), *Ae. tauschii *(2n = 14, DD), 7 000 to 12 000 years ago (for review [11]). Several wheat phylogeny studies have tried to identify the progenitor of the B genome of polyploid wheat based on cytology [12], nuclear and mitochondrial DNA sequences [13-15] as well as chromosome rearrangement studies (*i.e*. common translocation events) [16-24]. It remains controversial from those studies whether the progenitor of the B genome is a unique *Aegilops *species (*i.e*. monophyletic) or whether this genome resulted from an introgression of several parental *Aegilops *species (*i.e*. polyphyletic origin). More recent and representative molecular comparisons using germplasm collections have shown that the B genome could be related to several *Ae. speltoides *lines but not to other species of the *Sitopsis *section [25,26].

Transposable elements (TEs) have been shown since the seventies to be well represented in the wheat genome, ~80% [27,28]. Comparative studies have shown that beside the general conservation in coding sequences, no TE insertions are conserved between the A, B and D genomes of wheat whereas important proportion of TE insertions are shared between the A or D genomes of polyploid wheat and their respective progenitors *T. urartu *and *Ae. tauschii *[29-33]. No such studies have been yet reported comparing the B genome of these polyploid wheat species to that of its closest known diploid relative, *i.e*. *Ae. speltoides*. In the present study, we compared for the first time coding and non-coding sequences as well as dynamics of TE insertions between the S genome of *Ae. speltoïdes *and that of the A, B and D co-resident in the hexaploid wheat (*T. Aestivum*). The SPA (for Storage Protein Activator [34]) locus region, belonging to BZIP (Basic Leucine Zipper), located on chromosome 1BL [35], has been chosen because of its importance as trans-acting elements of seed storage protein and its conservation in several other cereals such as maize (Opaque 2 [36-38]), rice (RISBZ1-5 [39]), and barley (BLZ1-2 [40,41]). Updating phylogeny relationships and insights onto the origin of the B genome are discussed.

## Results

### Organization of SPA locus region in the A, B, D and S genomes

**Figure 1:**
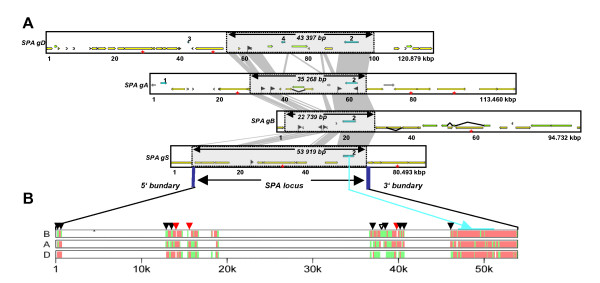
**Identification of the 'SPA orthologous region' and comparative annotation of the homoeologous A, B, D and S sequences**. (A) Scaled diagram of annotation results of the SPA locus region in which (CDS) (light blue), class I TEs (yellow blocks), class II TEs (green blocks), unclassified elements (grey), MITEs (vertical black flags) are shown. The remaining white spaces correspond to unassigned DNA (no features of annotation). Grey blocks represent sequence conservation between the different genomes defining the 'SPA orthologous region'. Genes are numbered as follow: 1: Pseudo tubulin gene; 2: SPA; 3: Putative cortical cell-delineating gene; 4: Putative kinesin gene. Eight class I TE displaying complete LTR and TSD suitable for the estimation of the insertion dates are highlighted with red stars. (B) Multipipmaker alignment using the sequence of the SPA orthologous region of the S genome of *Ae. speltoides *as a matrix compared with the 3 other sequences available, *i.e. T. aestivum *gB (top), gA (center), gD (bottom). Coloured blocks show the percentage of sequence identity (> 90 in red; between 50 to 90% in green). The SPA gene is indicated as a blue box.

Three BAC clones spanning the SPA gene of the A, B and D genomes of *T. aestivum *were screened from cv Renan BAC library with PCR markers specific for each of the three SPA genes [42]. Sequencing resulted in 113 460, 94 732 and 120 879 bp for, respectively, the A, B and D genomes. Screening of an *Ae. speltoides *pooled BAC library with the same SPA-specific PCR markers allowed us to identify and sequence a BAC clone of 80 493 bp sequence spanning the SPA locus gene. Annotation has been performed to identify and compare gene and repeat contents of the four available genome sequences, graphically presented in Figure 1A. More details are also presented in Additional File 1. As expected for wheat, the four genomic sequences are very rich in TEs.

Overall, the 113 460 bp A genome sequence is structured as 56 830 bp (50.1% of the sequence) of class I TE, 3 934 bp (3.5% of the sequence) of class II elements and 4.9% of unclassified TE. Fourteen class I TEs are identified as one incompletely sequenced (at the BAC sequence extremity), five truncated (with a 5' or 3' truncated region due to nested TE insertion), 4 relics (only visible through alignment remnants), one fragmented (inserted by other TEs, *i.e*. nested insertion) and three complete elements. The class II TEs is represented as a complete CACTA element (CACTA_1_comp, *cf *Additional File 2) and three MITEs (Miniature Inverted-repeat Transposable Element). Besides the identification of TEs a pseudo tubulin gene separated by 55 614 bp from the SPA gene was also identified, both genes covering 4.7% of the sequence.

The 94 732 bp B genome sequence is structured as 38 126 bp (40.2% of the sequence) of class I TEs, 22 602 bp (23.9% of the sequence) of class II elements and 0.6% of unclassified elements. Twelve Class I elements are identified as two incompletes, six truncated, two relics, one fragmented and one complete element. The class II TEs consists of two complete, one fragmented and one truncated CACTA (CACTA_1 to _4, *cf *Additional File 2) as well as three MITEs. The SPA gene is the only gene identified on the B genome sequence, representing 4.4% of the sequence.

The 120 879 bp D genome sequence is structured as 50 540 bp (41.8% of the sequence) of class I TEs, 9 446 bp (7.8% of the sequence) of class II elements. Twenty-two class I TEs are identified as two incomplete, eight truncated, eight relics, two fragmented and two complete elements. Class II TEs are represented as three truncated CACTA elements (CACTA_1 to 3, *cf *Additional File 2), one mutator relic and one MITE. Three genes have been annotated on the D genome sequence, the SPA gene, a putative kinesin and a putative cortical cell-delineating gene, covering 5.2% of a 48 440 bp interval.

The 80 493 bp S genome sequence is structured as 54 965 bp (68.3% of the sequence) of class I TEs, and a single MITE class II TE. Thirteen class I TEs are identified as one incomplete, six truncated, four fragmented and two complete TEs (*cf *Additional File 2). As in the B genome sequence, only the SPA gene, covering 4.3% of the annotated sequence, has been identified on the S genome sequence.

### Identification and characterization of conserved sequences

Alignment of the four genomic regions allows the identification of the 'SPA orthologous region', which we have defined as the shared common regions delimitated by conserved non-coding sequence (CNS) stretches (5' and 3' locus boundaries) that do not correspond to TEs. The 'SPA orthologous region' spans respectively 35 268 bp, 22 739 bp, 43 397 bp and 53 919 bp for the A, B, D and the S genomes (*cf *grey boxes in the Figure 1A, 1B).

**Figure 2:**
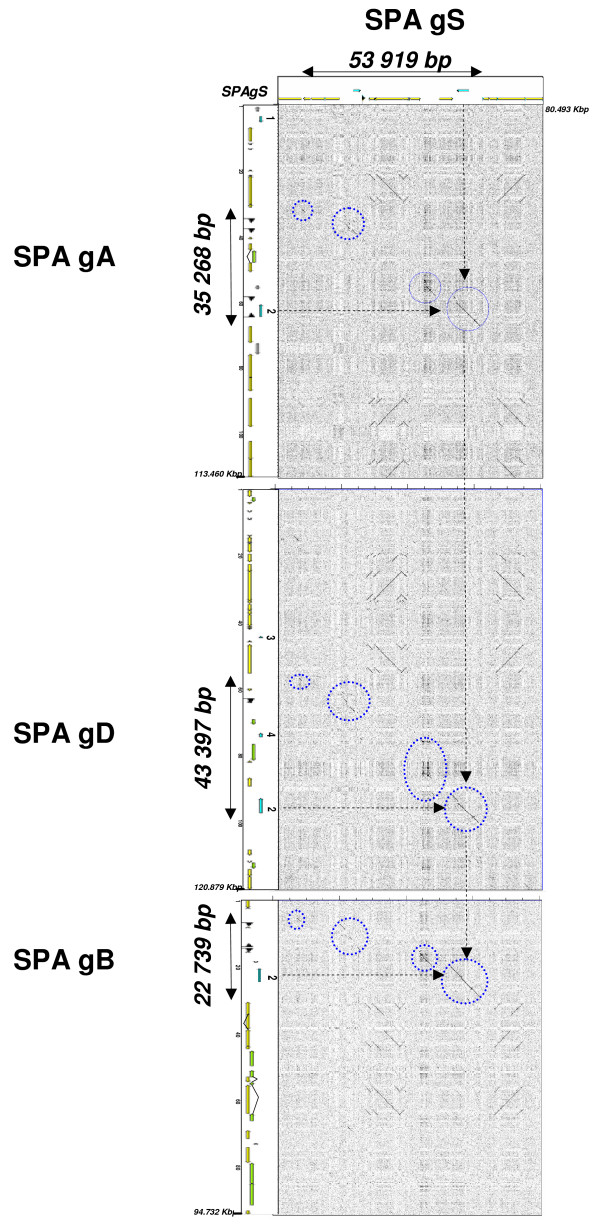
**Comparison of the Ae. speltoides sequence with the A, B D genome sequence of T. aestivum**. The dot plot was performed using the DOTTER program with default parameters between *Ae. speltoides *gS (horizontal) and *the T. aestivum *gA, -gB, -gD genome (vertical) sequences. Annotation features identified for these sequences are reported on the corresponding axes. Gene numbers and names as well as color codes for TEs and other DNA sequence classes are as in figure 1. Diagonals on the dot plot output that represent nucleotide conservation between the two analyzed sequences are highlighted with dotted blue circles. The loss of micro-colinearity corresponds to diagonal breaks. 'SPA orthologous region' defined as conserved sequences between *Ae. speltoides *gS and *T. aestivum *-gA, -gB, -gD sequences are mentioned with plain arrows on the four annotation features. SPA gene is shown with dotted arrows on the dot plot out put.

Dot plot analysis performed between *Ae. speltoides *gS (horizontal) and the *T. aestivum *gA, -gB, -gD genome (vertical) sequences, allows the identification of four conserved sequence stretches, highlighted by blue dotted circles in the Figure 2. The majority of the remaining DNA within the 'SPA orthologous region' (as well as outside the flanking boundaries) is composed of class I and class II TEs that were differentially inserted and/or deleted in each of the four genomes (*i.e*. shown by diagonal breaks on the dot plot in the Figure 2). The cumulative length of the conserved sequence stretches, within the 'SPA orthologous region' of the four genomes are approximately similar between the genomes pairs gA/gB (15 118 bp), gA/gD (14 677 bp), gA/gS (14 504 bp), gB/gD (14 628 bp), gB/gS(15 877 bp), gD/gS (13 985 bp). These could be considered as the *Aegilops*-*Triticum *'ancestral SPA Locus' covering 16 598 bp of cumulative length considering sequences stretches conserved between at least two of the compared sequence. Other stretches of sequence conservation were observed outside the 'SPA orthologous region' when comparing pairs of genomes but these sequences were not determined in the available BAC clone sequences of the other genomes (*data not shown*). As we cannot rule out whether these sequences were not covered in the sequenced BAC clones or were not really conserved across the four genomes, they were not considered in the evolutionary relationship analysis.

No genes, other than SPA can be predicted from these four conserved sequence stretches. As coding and non-coding sequences can evolve at different rates, we perform evolutionary analysis separately for the SPA CDS (CoDing Sequence) and the remaining conserved non-coding sequences (CNS).

#### Conserved non-coding sequences (CNS) analysis

The conserved non-coding sequences consist of the four shared sequence stretches, excluding the SPA gene itself (from methionine start to the stop codon). The gB/gS genome comparison shows the highest sequence identity and cumulative length (89.9% over 11 976 bp) compared to the other sequence comparisons, *i.e*. gA/gB (85.9% over 11 152 bp), gA/gD (87.9% over 10 838 bp), gA/gS (86.8% over 10 597 bp), gB/gD (85.8% over 10 666 bp), and gD/gS (85.3% over 10 039 bp) (*cf *Table 1). Nevertheless, only a 824 bp sequence was shown to be conserved between gS/gB (within the 11 976 bp of aligned sequence) and absent from other genomes (highlighted with white arrows in the Figure 1B). On the contrary, three sequence stretches (respectively 168, 340 and 218 bp) are conserved between the S, the A and/or D genomes and absent from the B genome (*cf *Figure 1B, red arrows). Moreover, although it represents the majority of the CNS comparisons, sequence conservation was not always the highest between the S and B genomes across the CNS as 9 small stretches (representing a total of 726 bp) of sequences were more conserved between the S and the A and/or D genomes than with the B genome (Figure 1B, black arrows).

**Table 1:** Conserved Coding (SPA gene) and Non-coding Sequences (CNS) identified between SPA-gA-gB-gD-gS at the 'SPA orthologous region'

**Non coding 'SPA orthologous loci' sequences**				**Coding 'SPA orthologous loci' sequences**			
	**B**	**D**	**S**		**B**	**D**	**S**

**A**				**A**			

**CNS size (bp)**	11 152	10 838	10 597	**Nb of transitions**	33	20	38

**% Identity**	85,9	87,9	86,8	**Nb of transversions**	22	10	23

**Ks**	0,874+- 0,036	1,037+-0,036	0,716+-0,024	**Ratio**	1,5	2	1,65

**Ka**	0,664+- 0,014	0,848+-0,015	0,57+-0,01	**Ks**	0,055+-0,015	0,042+-0,013	0,065+-0,016

**Ks/Ka**	1,3	1,2	1,3	**Ka**	0,042+-0,007	0,021+-0,005	0,049+-0,007

**B**				**MYA**	6,2–10,8	4,5–8,5	7,5–12,5

**CNS size (bp)**		10 666	11 976	**B**			

**% Identity**		85,8	89,9	**Nb de transitions**		35	25

**Ks**		0,991+-0,034	0,617+-0,026	**Nb de transversions**		20	19

**Ka**		0,797+-0,015	0,492+-0,012	**Ratio**		1,75	1,32

**Ks/Ka**		1,2	1,3	**Ks**		0,071+-0,017	0,035+-0,012

**D**				**Ka**		0,039+-0,007	0,035+-0,006

**CNS size (bp)**			10 039	**MYA**		8,3–13,5	3,5–7,2

**% Identity**			85,3	**D**			

**Ks**			0,902+-0,031	**Nb de transitions**			39

**Ka**			0,791+-0,014	**Nb de transversions**			23

**Ks/Ka**			1,1	**Ratio**			1,7

				**Ks**			0,089+-0,019

				**Ka**			0,043+-0,007

				**MYA**			10,8–16,6

***Non-coding 'SPA orthologous loci' sequences***. Detailed features obtained for the 6 pairwised alignments of the 4 SPA orthologous regions excluding the SPA gene itself are mentioned with the CNS length, percentage of sequence identity, Ks and Ka values and rate. As an example, alignment of the gA and gB SPA orthologous regions of respectively 31 498 bp and 18 589 bp correspond to a cumulative CNS length of 11 152 bp with 85.9% of sequence identity and 0.874, 0.664, 1.3 values for respectively Ks, Ka and Ks/Ka.

***Coding 'SPA orthologous loci' sequences***. 6 pairwised comparisons of the SPA gene between A/B B/D D/A A/S B/S D/S sequences are associated with the number of substitutions, the number of transition, the number of transversion, the transition/transversion ratio, the Ks value, the Ka value and speciation date (MYA).

We also estimated divergence times on the basis of the number of base substitutions (Ks) accumulated after the split-time from the ancestor genome. Ks values were obtained for the 6 pairwise alignment combinations (Table 1). The lowest and highest Ks values correspond respectively to the gB/gS (0.617, *i.e*. identifying the closest related sequences), and gB/gD (1.037, *i.e*. the more divergent sequences).

#### Conserved coding sequences analysis: SPA gene structure and evolution

SPA genes are structured as six exons (*cf *Additional File 2). In wheat, SPA gene (and CDS) are respectively 3 426(1 218) bp, 3 486(230) bp, 3 796(1 212) bp, 4 080(1 233) bp, long for A, B, D and S genes (hereafter designated SPAgA, -gB, -gD and -gS genes). These SPA genes are composed of six exons ranging in size from 76 (SPAgA, -gB, -gD, -gS exon 4) to 432 bp (SPAgA-gB-gS exon 1) and five introns ranging in size from 92 (SPAgA, -gB, -gD intron 4) to 1297 bp (SPAgD intron 5). All of exon-intron junction sites obey the GT/AG rule as identified in other eukaryotic genes. The relative organization of the exons and introns is the same for the others SPA-like bZIP protein genes characterized to date in cereal, *i.e*. the number of exons and introns is conserved and individual introns occur at relatively the same sites for the maize *O2 *[36-43], sorghum *O2 *[44], and barley *Blz1 *genes [40]. It is interesting to note that the first and fifth introns of the homoeologous SPA genes are respectively much shorter and larger, compare to the other cereal SPA-like bZIP protein genes (*cf *Additional File 2).

**Figure 3:**
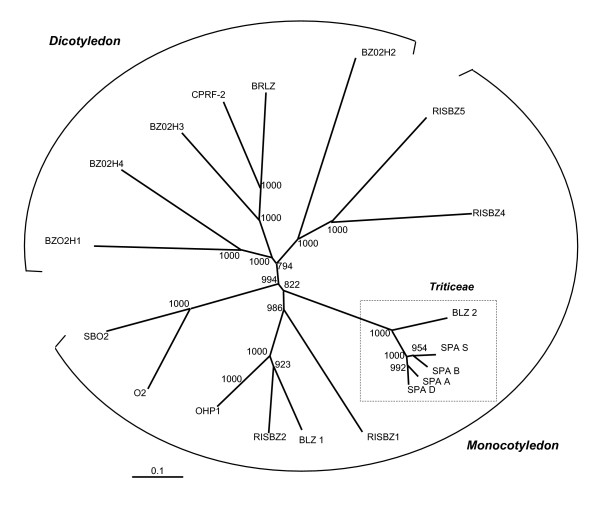
**Phylogenic analysis of the SPA protein among plant species**. 4 rice (RISBZ1-2-4-5 respectively AB053475, AB021736, AB053473, AB053474), 2 barley (BLZ1-2 respectively BLZ2, Y10834), 2 maize (O2-OHP1 respectively AJ491297, L00623), 1 sorghum (SBO2, X71636), 4 *Arabidopsis thaliana *(BZO2H1-4 respectively NM178959, NM122389, NM122760, NM115319), 1 *Nicotiana tabacum *(BRLZ, AY061648), 1 *Petroselinum crispum *(CPRF-2, X58577) and 4 wheat (SPA-gA, -gB, -gD, -gS, present analysis) sequences are involved in the tree. Parameters used to construct the tree are mentioned in the material and method section.

We conducted a phylogenic analysis based on SPA CDS of the four wheat genomes as well as that available from other cereals. A graphical representation of these data is shown in the Figure 3 with a classical phylogenic tree including SPA homologs available from other cereals (*cf *parameters in material and method) and illustrates that wheat SPA and barley BLZ2 consists in the same *Triticeae *subfamily in which *Ae. speltoides *and *T. aestivum*-gB SPA sequences are linked on the same branch. Such phylogenic analysis shows that the lowest synonymous (Ks) and non-synonymous (Ka) substitution rates were obtained between *Ae. speltoides *and *T. aestivum *-gB, with Ks (0.035+/-0.012) and Ka (0.035+/-0.006) values corresponding to a 3.5 to 7.2 MYA divergence time, while rates obtained when *Ae. speltoides *is compared to -gA and -gD are respectively Ks (0.065+/-0.016) and Ka (0.049+/-0.007) values corresponding to 7.5 to 12.5 MYA divergence time; and Ks (0.089+/-0.019) and Ka (0.043+/-0.007) values corresponding to 10.8 to 16.6 MYA divergence time (*cf *Table 1). This result strongly suggests that, despite the strong nucleotide conservation between the 3 homoeologous copies of the SPA CDS in *T. aestivum*, *Ae. speltoides *CDS is closest to the *T. aestivum *SPA-gB than the two other homoeologous -gA and -gD sequences.

As reported by Guillaumie et al. [35], a stop codon TGA (+19 bp from the ATG transcription initiation) site had been identified in the SPA-gB sequence suggesting that it might be no more functional. No proof of expression could be also provided for the SPA gB haplotype presenting this stop codon as we were unable to find any corresponding ESTs. In order to clarify the apparition of the TGA stop codon in the B genome, the stop codon allele distribution was analyzed using 18 wheat genotypes which cover, 1 diploid genome S (*Ae. longissima*), 11 tetraploid (3 *T. turgidum *durum, 3 *T. turgidum *dicoccoïdes, 2 *T. turgidum *dicoccum, 2 *T. timophevii*, 1 *T. turgidum turgidum*) and 6 hexaploid (*T. aestivum cv *soisson, arminda, vilmorin, chinese spring, renan, recital) genotypes. Genotyping data demonstrate that the TGA allele is present at 50% in hexaploid wheat (*T. cv soisson*, *vilmorin*, *renan*) and for the first time in one tetraploid (*T. turgidum durum*) genotype over 11 tested and absent in *Ae. longissima *(*cf *Additional file 3).

### Differential transposable elements insertions and evolution

Size discrepancies of the 'SPA orthologous regions' can be attributed to differential TE insertions or eliminations (*cf *Additional File 2 and Figures 1A and 2), which occurred after the four genomes divergence. Hence, the size increase observed for the 'SPA orthologous region' in *Ae. speltoides *(35 268 bp) when compared to *T. aestivum*-gB (22 739 bp) is due to 7 class I elements, *i.e*. 2 truncated Angela solo-LTRs (soloLTR_Angela_1 and _3), one complete Angela (Angela_2), one truncated Rada (Rada_1), 2 fragmented LINEs (LINE_1 and _2) and one MITE (*cf *Figure 2 and Additional File 2). These TEs may correspond to insertions, which occurred in the *Ae. speltoides *genome after its divergence from the ancestor of the B genome as they are dispersed between CNS stretches and not present in the B genome of *T. aestivum*. Occurrence of eight class I TEs displaying complete LTR and TSD (Target Site Duplication), identified in the four annotated genomes (highlighted with red stars in the Figure 1A) allows to estimate the insertion dates, based on nucleotide substitution pattern analysis (*cf *material and method; Additional File 4). Thus, the complete Angela_2 identified in *Ae. speltoides *(gS) located in the 'SPA orthologous region' exhibits a transition and tranversion value of 0.02 +/- 0.004 respectively associated with an estimated insertion time of 1.3 to 1.9 MYA. The youngest insertion time was observed for the Angela_5 element annotated outside the 'SPA orthologous region' in the *Ae. speltoides *sequence, *i.e*. 0.6 to 1.1 MYA.

## Discussion

We sequenced for the first time an *Ae. speltoides *genomic region (SPA locus region) and compared it to orthologous regions of the A, B and D genomes coresident in the hexaploid wheat *T. aestivum *at the SPA CDS, the CNS and the TE insertion dynamics levels.

### SPA gene structure comparison and haplotype variability

The SPA gene is the only gene conserved across the four genomes. A phylogenic analysis involving SPA protein sequences from *T. aestivum*, *Ae. speltoides*, rice, barley, maize, sorghum, *Arabidopsis thaliana*, *Nicotiana tabacum*, *Petroselinum crispum*, clearly identified a *Triticeae *outgroup in which *Ae. speltoides *SPA sequence is more closely related to *T. aestivum*-gB SPA than any other sequence involved in the tree. Interestingly, in this study we showed that the stop codon TGA allele, 19 bases downstream the ATG transcription initiation site, previously identified in the B genome of hexaploid wheat [42], is also present in the tetraploid *T. turgidum*. This indicates that the stop-codon TGA SPA allele has been generated before the allohexaploidization event. The presence of both stop TGA and TCA SPA alleles in tetraploid and hexaploid wheat accessions provides further evidences for the hypothesis of (i) recurrent hexapolyploidization events or (ii) gene flow through introgression between the different wheat species with different ploidy levels [30-33].

### Differential pattern of CNS conservation

Our results reveal that, a large proportion of the remaining non-genes and non-transposable elements sequences are highly conserved between the four genomes (CNS). At the 'SPA orthologous region', excluding the SPA gene itself, the gB/gS genome comparison shows the highest sequence identity and cumulative length as well as the lowest Ks value (89.9% over 11 976 bp with Ks = 0.617) compared to the other sequences (*cf *Table 1). Thus, the S genome was confirmed to be the closest to the B genome in term of cumulative conserved sequence length as well as identity as compared to any other pairwise genome combinations. Small stretches of sequences, which were more conserved between the S and/or the A and D genomes (*cf *Figure 1B), do not contradict with the general pattern of an overall higher CNS conservation between the S and B genomes. This is the first time that we precisely report close relationships between the S and B genomes based on both coding and non-coding sequence comparisons. CNS (within introns or upstream regulatory sequences), have been recently surveyed in cereals (maize *vs *rice) and mammals (human *vs *mouse) [45,46]. It has been shown that CNSs are more abundant in loci embedding regulatory genes such as transcription factors (as SPA gene described in our study) and that despite divergence from a common ancestors, grass genes have dramatically fewer (5- to 20-fold) and smaller CNSs than mammalian genes. One possible explanation is that, in contrast to vertebrate genomes, plant genomes have been subjected to more rounds of whole genome duplications (polyploidization) events that have profoundly affected their organisation, the subfunctionalisation of duplicated genes leading to a greater per gene loss of CNS [47].

### Differential TE insertion dynamics

**Figure 4:**
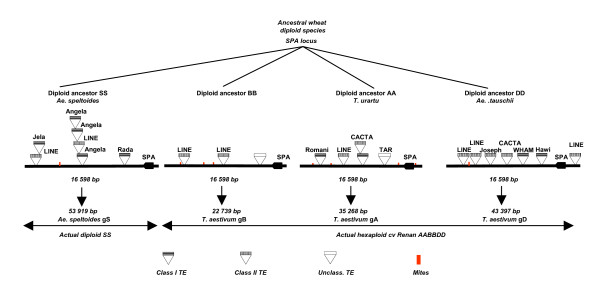
**Evolutionary structure of the 'Ancestral SPA Locus'**. From the 'Ancestral SPA Locus' of 16 598 bp, nested insertions of identified TE are shown for the four sequences (gA, gB, gD, gS). Graphical motifs used to materialize class I, class II unclassified TE as well as MITE are mentioned on the figure.

No class I or class II TE annotated within or outside the 'SPA orthologous region' is common when comparing any two-genome combinations. The two *WIS *retrotransposons, displaying similar apparent insertion positions in the 5' SPA locus boundaries of the A and D genomes correspond to independent insertions as Target Site Duplication (TSD) signature-motifs are distinct (respectively TATTG and TGTGA). This is also confirmed by estimation of their insertion dates with a transition and transversion ratio of 0.0029+/-0.004 (*i.e*. insertion date of 1.9–2.6 MYA) and 0.012+/-0.003 (*i.e*. insertion date of 0.7–1.2 MYA) for respectively the A and D genome sequences (*cf *Additional File 4). The differential insertion of TEs is surprisingly the case of the B and S genomes. Overall, we count six (two class II TEs, one unclassified TE and three MITEs) and eight (five class I TEs, two class II TEs and one MITE) TEs differentially inserted in the B and S genomes respectively (*cf *Figure 1A). The 'SPA orthologous region' of the S genome has been invaded by retrotransposons, whereas outside the 'SPA orthologous region' the B genome seems to have a specific site for the insertion of class II TEs (mainly CACTA elements representing 23.9% of the sequence). Overall, we were able to estimate insertion dates for 8 retrotransposons. Out of them, only one (Angela_2) has been inserted into the 'SPA orthologous region' of *Ae. speltoides*, (estimated insertion date 1.3 to 1.9 MYA). Thus, the differential insertions of TEs in the S genome might be posterior to the S and B genome progenitors divergence from a common ancestor 2.5 MYA, 3.5 in the present study. Figure 4 retraces the process of TE differential insertion-deletions from a suggested *Triticum-Aegilops *'ancestral SPA Locus' sequence of 16 598 bp that has been subjected to intensive TE insertions in the A, D and S genomes as compared to the B genome analysed in the present study.

### The progenitor enigma of the B genome of polyploid wheat species

According to the two allopolyploidization events that gave rise to *T. aestivum*, the D genomes of the hexaploid wheat have diverged relatively recently from that of its donnor *Ae. tauschii *(0.08–0.12 MYA) whereas divergence of the A and B genomes from their respective progenitors occurred much more earlier (< 0.5 MYA) [7,9,10]. For almost 50 years, it remained controversial whether the source of the B genome is unique (*i.e*. monophyletic origin) related to *Ae. speltoides *or whether this genome resulted from an introgression of several parental *Aegilops *species (*i.e*. polyphyletic origin) [9,12-24,48]. Recent data on molecular comparisons using germplasm collections clearly show that the B genome could be related to several *Ae. speltoides *lines but not to other species of the Sitopsis section [25,49].

Comparison between the A genome of polyploid wheat species to that of its progenitor *T.urartu *at the PSR920 region [32] has shown a very high CDS conservation (99.5% of sequence identity at the third base of codons and 99.6% for introns). Moreover, Dvorak et al. [32] found in the 103 kb intergenic sequences four conserved TEs (inserted prior to their divergence) whereas four and one other TEs were respectively inserted in the A genome of *T. urartu *and that of *T. durum*, after their divergence from a common ancestor. Our present comparison based on CDS and CNS confirms that the B genome is closer to the S genome of *Ae. speltoides *than the A and D genomes. However, SPA sequence divergence and the differential insertions/deletions of TEs, none of which is conserved between the two genomes, indicate that *Ae. speltoids *have diverged very early (> 3MYA, in our study) from the B genome progenitor.

## Conclusion

The present study based on detailed CDS, CNS and TE dynamics comparisons, clearly shows that evolutionary relationship between the B genome and the S genome of *Ae. speltoides *is not as close as it has been reported in the literature for the A genome of polyploid wheat species compared to its identified progenitor, *T. urartu*. Thus, a B genome progenitor remains to be identified.

## Methods

### BAC Clone Isolation

A BAC (Bacterial Artificial Chromosomes) library from *T. aestivum *cv renan [50] and *Ae. speltoides *BAC library (Chalhoub et al., unpublished) were screened with SPA PCR markers [34,42]. Assignment to the A, B, or D genomes of the BAC clones from the hexaploid species was based on their further characterization by Hind*III *restriction fragment length polymorphisms and specific PCR primers [42]. To ensure maximum coverage of the SPA locus, the longest BAC clones for the A (Ren1424A05, Accession#: FM242575), B (Ren0871J20, Accession#: FM242576), D (Ren2409K09, Accession#: FM242578) and S (Sho42-9K3, Accession#: FM242577) genomes were sequenced.

### BAC sequencing and annotation

BAC shotgun sequencing was performed at the Centre National de Sequencage (Evry, France). Genes and repeated elements (TEs and short repeats) were identified by computing and integrating results based on BLAST algorithms [51,52], predictor programs, and different software detailed as follows.

#### Gene structure analysis

Gene structures and putative functions were identified by combining results of BLASTN and BLASTX alignments against dbEST  and SwissProt databases , with results of 2 gene predictor programs, Eugene [53] with rice (*Oryza sativa*) training version and FgeneSH [54] (with default parameters . To incorporate heterologous information, we only recovered potential gene coding sequences. The CDS (CoDing Sequence) structures correspond to a consensus derived from the three preceding information sources. The gene content parameter represents the sum of known genes, hypothetical genes, unknown genes, and pseudogenes. Known genes were named based on BLASTX results against proteins with known functions (SwissProt). CDSs were considered as (i) hypothetical genes if their identification was only based on the predictors (as a consensus of the structures suggested by both predictors), without any evidence of putative function based on BLASTX results; (ii) unknown genes if the identification was only based on matching ESTs, without any evidence of putative function based on BLASTX results; (iii) pseudogenes if frame shifts need to be introduced within the CDS structure to better fit a putative function based on BLASTX results. Truncated pseudogenes, (genes disrupted by large insertion or deletion) and highly degenerated CDS sequences were considered as gene relics.

#### Transposable elements (TE)

TEs were detected by comparison with two databases of repetitive elements: TREP ([55]; ), and Repbase ([56]; ). Core domains (nucleic coordinates of known elements) were identified through BLASTN alignments against TREPnr. LTRs (Long Terminal Repeats) and TE boundaries were identified through BLASTN alignments against Repbase. Putative polyproteins were identified by BLASTX alignments against TREPprot. We used 1e^-04 ^as a cutoff for BLASTN alignment results (either on TREPnr or Repbase). No cut-off was imposed for BLASTX results on TREPprot. Nested insertions of TEs were considered only when complete reconstruction of the split element was possible with no ambiguity. Other TE structures (either novel or highly degenerated TEs) were identified within the remaining unassigned DNA either by LTR_STRUC [57] or by BLASTX against the NCBI nr database . When it was possible (*i.e*. for complete TEs), target-site duplications were indicated in the commentary of the element.

Pairwise comparisons of the four BAC clones, including the analysis of each BAC sequence against itself, were performed using the program Dotter [58] in order to identify or confirm direct repeats, LTRs, local duplications, and deletion events as well as MITEs. Multiple sequences comparisons were performed with PIPMAKER software [59]. As a final screening, unassigned DNA (free of annotated genes or TEs) was aligned using BLASTX against the NCBI nonredundant database . This BLASTX analysis allows the extension of several TE features already identified. TEs were classified and named based on the unified classification from Wicker et al. [60] according to referred nomenclature (*i.e*., element name, BAC name, appearance rank) and designed as complete, truncated, and degenerated sequences as suggested by TREP or Repbase databases.

#### Short repeated motifs

Short repeated motifs were identified either as inverted repeats (by using EINVERTED with default parameters; ) or tandem repeats (Tandem Repeat Finder, with default parameters; ). Only repeated domains (*i.e*. tandem or inverted) longer than 100 bp were kept in our annotation results.

#### Unassigned DNA sequences

Unassigned DNA corresponds to sequences in which neither CDS nor TE was identified. Such unassigned DNA may contain short repetitive units (tandem repeats or inverted repeats).

#### Integration of annotation results

Cross-analysis of the information obtained for genes and TEs as short repeats was integrated into ARTEMIS [61].

### Sequence analysis

#### Multiple alignments

Identification of conserved domains was performed based on multiple alignments (clustalw, [62]) on translated SPA CDS (identified from the sequence annotation procedure).

#### Phylogeny analysis

The phylogenetic analysis was performed using Neighbor-joining method with clustalx alignment of protein sequences with 1 000 repetition bootstraps. The BLOSUM 62 matrix was chosen for substitution identification. The sequence divergence datation was performed based on the rate of nonsynonymous (*Ka*) vs. synonymous (*Ks*) substitutions calculated with MEGA-3 [63]. The average substitution rate (r) of 6.5 × 10^-9 ^substitutions per synonymous site per year for grasses was used to calibrate the ages of the considered gene ([64,65]. The time (*T*) since gene insertion was estimated using the formula *T *= *Ks*/r.

### Determination retrotransposons insertion dates

Full-length retrotransposons were analysed by comparing their 5' and 3' LTR sequences in order to date their insertion time [65] based on the assumption that the two LTRs of a single element are identical at the time of insertion. The two LTRs were aligned and the number of transition and transversion mutation were counted. The insertion times were dated using the Kimura parameter method (K2P, [66]) and a mutation rate of 6.5 × 10^-9 ^substitutions per synonymous site per year [64]. The time (*T*) since element insertion was estimated using the formula T = *K2P/2r*.

## Authors' contributions

JSperformed the BAC sequence annotation and analysis and comparative annotation and wrote the manuscript. VC*, *SB*, *CP*, *MC and NB contributed in sequence analysis and annotation as well as transposable elements evolution. CH*, *HB*, *SG and AEwere implicated into *(i) *the construction of the *Ae. speltoides *BAC library, *(ii) *the screening of the BAC libraries, *(iii) *the identification and verification of the positive BAC clones as well as PCR genotyping. GM*, *AC*, *BSand SSwere implicated in BAC clone sequencing, sequence assembly and verification of assembled sequences. CRand GCwere involved in the interpretation of SPA gene sequence comparisons. BC**, **coordinator of the project, set up the project and followed analysis and interpretation of the results as well as wrote and edited the manuscript.

## Supplementary Material

Additional file 1 **BAC clones annotation**. detailed annotation features for T. aestivum -gA, -gB, -gD and Ae. speltoides gS sequences as GenBank format files. Click here for file

Additional file 2 **BAC clones gene and TE content**. Detailed features (genes, TE) of the 4 annotated BAC clone T. aestivum -gA, -gB, -gD and Ae. speltoides gS sequences. Click here for file

Additional file 3 **SPA genotyping data**. SPA genotyping data among 18 wheat genotypes. Click here for file

Additional file 4 **TE divergence analysis**. Divergence time for 8 complete Class I transposable elements. Click here for file
