# Supplementary material for: New insights into the origin of the B genome of hexaploid wheat: Evolutionary relationships at the SPA genomic region with the S genome of the diploid relative Aegilops speltoides
Source: BMC Genomics. 2008 Nov 25;9:555. doi: 10.1186/1471-2164-9-555 (PMC2612700; doi:10.1186/1471-2164-9-555)
Supplement: Additional file 1 — BAC clones annotation. detailed annotation features for T. aestivum -gA, -gB, -gD and Ae. speltoides gS sequences as GenBank format files. [file 1471-2164-9-555-S1.doc]

***Supplementary File 1 -*** Detailed annotation features for *T. aestivum* –gA, -gB, -gD and *Ae. speltoides* gS sequences

**Supplementary File 1-1: T. aestivum gA annotation file**

ID ENTRYNAME SPA genome A; DNA; PLN; 113460 BP.

XX

HD * confidential

XX

AC * not submitted

AC ;

XX

DE Triticum sp.

CC Sequencing was performed at Genoscope and annotations were obtained by

CC Salse Jerome, Charles Mathieu.

XX

OS Triticum sp

OC Eukaryota; Viridiplantae; Streptophyta; Embryophyta; Tracheophyta;

OC Euphyllophyta; Spematophyta; Magnoliophyta; Liliopsida; Poales; Poaceae;

OC Pooideae; Triticeae; triticum aestivum cv renan.

XX

XX

FH Key Location/Qualifiers

FT repeat_region complement(3..1337)

FT /note="Unclass-1 incomplete"

FT gene 3000..4823

FT /note="speudo Tubulin"

FT CDS join(3202..3486,3822..3928,4020..4395,4767..4823)

FT /note="speudo Tubulin"

FT mRNA join(3202..3486,3822..3928,4020..4395,4767..4823)

FT /note="speudo Tubulin"

FT LTR complement(6341..8135)

FT /note="LTR_LAURA_1 truncated""

FT repeat_region complement(6341..10526)

FT /note="LAURA_1 truncated"

FT repeat_region join(11532..11653,13028..13189)

FT /note="LINE_1_relic"

FT repeat_region complement(19272..19648)

FT /note="LINE_2_relic"

FT LTR complement(join(20846..21190,29775..30826))

FT /note="soloLTR_WHAM_1_truncated"

FT LTR complement(join(21193..22871,28037..29774))

FT /db_xref="WIS2_TM_LTR long terminal repeat. (1776 bp)"

FT /note="LTR_WIS_1_complete"

FT repeat_region complement(21193..29774)

FT /note="WIS_1_complete"

FT misc_feature complement(join(23177..23227,23232..23348,23336..23725,23715..23795,23797..23877,23864..23959,23956..23988,23991..24551,24586..24780,24783..25088,25085..25732,25735..25911,25931..26005,26082..26138,26188..26373,26372..26698,26697..26717))

FT /note="WIS_1_complete"

FT repeat_region 34215..34267

FT /note="MITE"

FT repeat_region complement(35078..35296)

FT /note="Romani_1_relic"

FT repeat_region 37306..37390

FT /note="MITE"

FT repeat_region complement(39874..40459)

FT /note="LINE_3_relic"

FT repeat_region join(41983..44007,47687..50465)

FT /note="TAR_1_fragmented"

FT misc_feature join(42281..42460,42454..42855,42942..43265,43345..44007,47687..47893,47862..48869,48863..49399,49402..49452,49452..49625)

FT /note="TAR_1_fragmented"

FT repeat_region complement(44006..47683)

FT /note="CACTA_1 complete"

FT repeat_region 54789..55788

FT /note="Unclass_2_relic"

FT repeat_region 58051..58128

FT /note="MITE"

FT gene complement(60437..64206)

FT /note="SPA"

FT CDS complement(join(60655..61035,61671..61796,61889..61964,62636..62756,62932..63013,63649..64080))

FT /note="SPA"

FT mRNA complement(join(60655..61035,61671..61796,61889..61964,62636..62756,62932..63013,63649..64080))

FT /note="SPA"

FT repeat_region complement(64289..64328)

FT /note="MITE"

FT LTR 67275..68822

FT /note="LTR_SABRINA_1_truncated"

FT repeat_region 67275..72382

FT /note="SABRINA_1_truncated"

FT repeat_region 72426..75658

FT /note="Unclass_3_truncated"

FT repeat_region complement(75714..82817)

FT /note="SABRINA_2_complete"

FT LTR complement(join(75714..77284,81237..82817))

FT /note="LTR_SABRINA_2_complete"

FT repeat_region 82902..87158

FT /note="JELI_1_truncated"

FT LTR join(89274..91003,96316..98047)

FT /note="LTR_WIS_2_complete"

FT repeat_region 89274..98047

FT /note="WIS_2_complete"

FT misc_feature 92309..96280

FT /note="WIS_2_complete"

FT CDS complement(join(99123..99535,99629..100048,100181..100375,100484..100738))

FT /note="Hypothetical gene"

FT gene complement(99123..100738)

FT /note="Hypothetical gene"

FT mRNA complement(join(99123..99535,99629..100048,100181..100375,100484..100738))

FT /note="Hypothetical gene"

FT repeat_region 102309..107924

FT /note="SABRINA_3_truncated"

FT LTR join(102309..103949,107590..107924)

FT /note="LTR_SABRINA_3_truncated"

FT LTR 107925..109656

FT /note="LTR_WIS_3_incomplete"

FT repeat_region 107925..113460

FT /note="WIS_3_incomplete"

FT misc_feature join(110989..111066,111078..111293,111286..111402,111411..111566,111601..111699,111693..111830,111832..112017,112054..112182,112173..112310,112349..112717,112719..113051,113087..113341,113344..113460)

FT /note="WIS_3_incomplete"

**Supplementary File 1-2: T. aestivum gB annotation file**

ID ENTRYNAME SPA genome B; DNA; PLN; 94732 BP.

XX

HD * confidential

XX

AC * not submitted

AC ;

XX

DE Triticum sp.

CC Sequencing was performed at Genoscope and annotations were obtained by

CC Salse Jerome, Charles Mathieu.

XX

OS Triticum sp

OC Eukaryota; Viridiplantae; Streptophyta; Embryophyta; Tracheophyta;

OC Euphyllophyta; Spematophyta; Magnoliophyta; Liliopsida; Poales; Poaceae;

OC Pooideae; Triticeae; triticum aestivum cv renan.

XX

XX

FH Key Location/Qualifiers

FT repeat_region 1..2618

FT /note="BAGY_1_incomplete"

FT LTR 1068..2618

FT /note="LTR_BAGY_1_incomplete"

FT LTR complement(2636..2752)

FT /note="soloLTR_BARBARA_1_truncated"

FT repeat_region 7128..7160

FT /note="MITE"

FT repeat_region complement(8314..8484)

FT /note="LINE_1_relic"

FT repeat_region complement(14338..14424)

FT /note="MITE"

FT repeat_region 14853..15139

FT /note="MITE"

FT repeat_region complement(15804..15929)

FT /note="LINE_2_relic"

FT repeat_region 18972..19190

FT /note="unclassified_1_truncated"

FT gene complement(20866..25015)

FT /note="SPA"

FT CDS complement(join(21107..21490,22128..22253,22346..22421,23122..23242,23755..23845,24471..24902))

FT /note="SPA"

FT mRNA complement(join(21107..21490,22128..22253,22346..22421,23122..23242,23755..23845,24471..24902))

FT /note="SPA"

FT LTR complement(join(30929..32708,42366..44129))

FT /note="LTR_WIS_1_fragmented"

FT repeat_region complement(join(30929..34716,39234..44129))

FT /note="WIS_1_fragmented"

FT misc_feature complement(join(32903..32950,33010..33120,33137..33193,33196..33402,33414..33818,33881..34065,34068..34196,34199..34481,34492..34605,34611..34739,39234..39344,39356..39472,39535..39636,39644..39715,39754..39891,39909..40010,40053..40205,40207..40335,40338..40436,40471..40626,40635..40750,40752..40842,40962..41075))

FT /note="WIS_1_fragmented"

FT LTR 34714..39148

FT /note="soloLTR_SUKKULA_1_truncated"

FT repeat_region complement(44130..45122)

FT /note="WIS_2_truncated"

FT misc_feature complement(join(44324..44371,44431..44541,44558..44821,44833..45111))

FT /note="WIS_2_truncated"

FT repeat_region complement(45631..50215)

FT /note="CACTA_1_truncated"

FT misc_feature complement(join(46234..46341,46466..46549,46807..46917,47006..47134,47134..48015,48212..49723,49889..50095))

FT /note="CACTA_1_truncated"

FT repeat_region complement(join(51464..53517,55102..55886,64540..66671))

FT /note="CACTA_2_fragmented"

FT misc_feature complement(join(53167..53535,55101..55787,55789..55851,55842..55886,64540..64746,64719..64985,65088..65180,65162..65275,65270..65362))

FT /note="CACTA_2_fragmented"

FT LTR 53538..55105

FT /note="soloLTR_SABRINA_1_truncated"

FT LTR complement(join(55887..57631,62795..64535))

FT /note="LTR_WIS_3_complete"

FT repeat_region complement(55887..64535)

FT /note="WIS_3_complete"

FT misc_feature complement(join(57826..57873,57933..58064,58040..58324,58336..58776,58746..59291,59406..59543,59574..59732,59762..59923,59923..60024,60032..60103,60142..60276,60297..60596,60623..60760,60754..61035,61050..61154,61159..61257,61376..61489))

FT /note="LTR_WIS_3_complete"

FT repeat_region complement(69568..72095)

FT /note="Yvonne_1_truncated"

FT repeat_region complement(73325..73720)

FT /note="unclassified_2_truncated"

FT repeat_region 74559..79200

FT /note="Ale_1_truncated"

FT repeat_region complement(79348..85501)

FT /note="CACTA_3_complete"

FT misc_feature complement(join(80027..80224,80320..80391,80462..80608,80719..80907,80994..81560,81578..81637,81702..81770,82200..82424,82430..83167,83134..83673,83732..83950))

FT /note="CACTA_3_complete"

FT repeat_region complement(85501..91985)

FT /note="CACTA_4_complete"

FT misc_feature complement(87355..90684)

FT /note="CACTA_4_complete"

FT repeat_region 93663..94732

FT /note="Romani_1_incomplete"

**Supplementary File 1-3: T. aestivum gD annotation file**

ID ENTRYNAME SPA genome D; DNA; PLN; 120879 BP.

XX

HD * confidential

XX

AC * not submitted

AC ;

XX

DE Triticum sp.

CC Sequencing was performed at Genoscope and annotations were obtained by

CC Salse Jerome, Charles Mathieu.

XX

OS Triticum sp

OC Eukaryota; Viridiplantae; Streptophyta; Embryophyta; Tracheophyta;

OC Euphyllophyta; Spematophyta; Magnoliophyta; Liliopsida; Poales; Poaceae;

OC Pooideae; Triticeae; triticum aestivum cv renan.

XX

XX

FH Key Location/Qualifiers

FT repeat_region 43..2042

FT /note="Fatima_1_incomplete"

FT repeat_region 2320..3560

FT /note="CACTA_1_truncated"

FT LTR 3779..4336

FT /note="SoloLTR_FATIMA_2_tuncated"

FT repeat_region 6624..6788

FT /note="LINE_1_relic"

FT repeat_region 8941..9024

FT /note="Angela_1_relic"

FT repeat_region join(13802..14185,15972..16298,41864..42289)

FT /note="Unclass_1_fragmented"

FT LTR complement(14192..15786)

FT /note="soloLTR_BARBARA_1_truncated"

FT LTR join(16334..18848,41286..41852)

FT /note="SABRINA_1_fragmented"

FT LTR 19399..20485

FT /note="LTR_ANGELA_2_truncated"

FT repeat_region 19399..21767

FT /note="ANGELA_2_truncated"

FT repeat_region 22489..24719

FT /note="LTR_SABRINA_2_fragmented"

FT LTR join(22489..23940,34469..36422)

FT /note="LTR_SABRINA_2_fragmented"

FT LTR join(24721..26466,31721..33561)

FT /note="LTR_ANGELA_3_complete"

FT repeat_region 24721..33561

FT /note="ANGELA_3_complete"

FT misc_feature join(27717..27983,27983..28522,28518..30923,30884..31684)

FT /note="ANGELA_3_complete"

FT LTR 33560..34365

FT /note="LTR_ANGELA_4_truncated"

FT repeat_region 36511..37701

FT /note="Hawi_1_truncated"

FT LTR 37760..38781

FT /note="LTR_SABRINA_3_truncated"

FT repeat_region 37760..41287

FT /note="LTR_SABRINA_3_truncated"

FT misc_feature join(39303..39608,39602..40150)

FT /note="LTR_SABRINA_3_truncated"

FT mRNA complement(44244..44815)

FT /note="Putative Cortical cell delineating gene"

FT gene complement(44244..44815)

FT /note="Putative Cortical cell delineating gene"

FT CDS complement(44424..44768)

FT /note="Putative Cortical cell delineating gene"

FT repeat_region complement(45578..46014)

FT /note="Yvonne_1_relic"

FT LTR complement(join(46688..48403,53674..55388))

FT /note="LTR_WIS_1_complete"

FT repeat_region complement(46688..55388)

FT /note="WIS_1_complete"

FT misc_feature complement(48439..52359)

FT /note="WIS_1_complete"

FT repeat_region 60127..60735

FT /note="LINE_2_relic"

FT repeat_region 63107..63142

FT /note="MITE"

FT repeat_region complement(63824..63997)

FT /note="LINE_3_relic"

FT repeat_region 69425..70988

FT /note="Joseph_1_relic"

FT CDS complement(join(73577..74053,74090..74230,74528..74671,74761..74838))

FT /note="Putative Kinesin heavy chain isoform gene"

FT gene complement(73577..74838)

FT /note="Putative Kinesin heavy chain isoform gene"

FT mRNA complement(join(73577..74053,74090..74230,74528..74671,74761..74838))

FT /note="Putative Kinesin heavy chain isoform gene"

FT repeat_region 76901..81866

FT /note="CACTA_2_truncated"

FT repeat_region complement(81866..82393)

FT /note="WHAM_1_relic"

FT repeat_region complement(87059..89603)

FT /note="Hawi_2_relic"

FT gene complement(93255..97726)

FT /note="SPA"

FT CDS complement(join(93474..93854,95152..95277,95370..95445,96123..96243,96421..96511,97137..97553))

FT /note="SPA"

FT mRNA complement(join(93474..93854,95152..95277,95370..95445,96123..96243,96421..96511,97137..97553))

FT /note="SPA"

FT repeat_region 108810..110495

FT /note="LINE_4_relic"

FT repeat_region 112583..112812

FT /note="Angela_5_truncated"

FT misc_feature join(112817..113173,113285..113827)

FT /note="CACTA_3_truncated"

FT repeat_region 112817..114455

FT /note="CACTA_3_truncated"

FT repeat_region 114771..116978

FT /note="Claudia_1_truncated"

FT misc_feature join(116118..116210,116253..116735,116904..116978)

FT /note="Claudia_1_truncated"

FT misc_feature 116979..120879

FT /note="Inga_1_incomplete"

**Supplementary File 1-4: Ae. speltoides gS annotation file**

ID ENTRYNAME SPA genome S; DNA; PLN; 80493 BP.

XX

HD * confidential

XX

AC * not submitted

AC ;

XX

DE Triticum sp.

CC Sequencing was performed at Genoscope and annotations were obtained by

CC Salse Jerome, Charles Mathieu.

XX

OS Triticum sp

OC Eukaryota; Viridiplantae; Streptophyta; Embryophyta; Tracheophyta;

OC Euphyllophyta; Spematophyta; Magnoliophyta; Liliopsida; Poales; Poaceae;

OC Pooideae; Triticeae; Aegilops speltoides.

XX

XX

FH Key Location/Qualifiers

FT repeat_region complement(1..7139)

FT /note="Barbara_1_incomplete"

FT repeat_region complement(join(7871..9828,14281..18786))

FT /note="LINE_1_fragmented"

FT repeat_region 10030..14187

FT /note="Jela_1_fragmented"

FT CDS join(22907..23012,23133..23328,24633..24873)

FT /note="hypothetical gene"

FT gene 22907..24873

FT /note="hypothetical gene"

FT mRNA join(22907..23012,23133..23328,24633..24873)

FT /note="hypothetical gene"

FT repeat_region 25499..25610

FT /note="MITE"

FT LTR complement(27604..29294)

FT /note="soloLTR_ANGELA_1_truncated"

FT repeat_region join(29295..29648,40005..43161)

FT /note="LINE_2_fragmented"

FT repeat_region complement(join(29630..37867,39631..40013))

FT /note="ANGELA_2_complete"

FT LTR complement(join(29630..31392,36481..37867,39631..40013))

FT /note="LTR_ANGELA_2_complete"

FT misc_feature complement(31694..35242)

FT /note="ANGELA_2_complete"

FT LTR 37868..39630

FT /note="soloLTR_ANGELA_3_truncated"

FT repeat_region 48852..52851

FT /note="Rada_1_truncated"

FT CDS complement(join(54520..54906,55549..55674,55769..55844,56552..56672,56854..56944,57574..58005))

FT /note="SPA"

FT gene complement(54520..58005)

FT /note="SPA"

FT mRNA complement(join(54520..54906,55549..55674,55769..55844,56552..56672,56854..56944,57574..58005))

FT /note="SPA"

FT LTR 61980..62211

FT /note="soloLTR_ANGELA_4_truncated"

FT LTR 62381..63965

FT /note="soloLTR_INGA_1_truncated"

FT repeat_region 64028..66521

FT /note="Inga_2_truncated"

FT LTR join(66522..68276,73366..75126)

FT /note="LTR_ANGELA_5_complete"

FT repeat_region 66522..75126

FT /note="ANGELA_5_complete"

FT misc_feature 69517..73175

FT /note="ANGELA_5_complete"

FT repeat_region 75127..80368

FT /note="Inga_3_fragmented"

FT misc_feature join(75241..76495,76539..76741,76855..77482,77629..78499,78933..79146)

FT /note="Inga_3_fragmented"
